# A multicenter study of interocular symmetry of corneal biometrics in Chinese myopic patients

**DOI:** 10.1038/s41598-021-84937-9

**Published:** 2021-03-10

**Authors:** Guihua Xu, Yijun Hu, Shanqing Zhu, Yunxiang Guo, Lu Xiong, Xuejun Fang, Jia Liu, Qingsong Zhang, Na Huang, Jin Zhou, Fangfang Li, Xiaohua Lei, Li Jiang, Zheng Wang

**Affiliations:** 1 Department of Ophthalmology, Huizhou Municipal Central Hospital, Huizhou, China; 2 Aier Institute of Refractive Surgery, Refractive Surgery Center, Guangzhou Aier Eye Hospital, Guangzhou, China; 3 Aier School of Ophthalmology, Central South University, Fourth Floor, New Century Mansion, 198 Middle Furong Road, Changsha, 410015 China; 4 Refractive Surgery Center, Shenyang Aier Eye Hospital, Shenyang, China; 5 Refractive Surgery Center, Wuhan Aier Eye Hospital, Wuhan, China; 6 Refractive Surgery Center, Chengdu Aier Eye Hospital, Chengdu, China; 7 Refractive Surgery Center, Hankou Aier Eye Hospital, Hainan, China

**Keywords:** Diagnostic markers, Diagnostic markers

## Abstract

It is essential to know the normal range of the interocular symmetry of the cornea (ISC) for keratoconus diagnosis and corneal substitutes design. In the present study we investigated the interocular symmetry of corneal biometrics in 6,644 Chinese myopic patients from multiple ophthalmic centers. Corneal biometrics of both eyes were exported from the Pentacam instrument. Interocular symmetry of the corneal biometrics was analyzed by Spearman’s correlation test, intraclass correlation coefficient (ICC) analysis and Bland–Altman plot. Significantly strong interocular correlations were found in anterior and posterior corneal curvatures, corneal diameter, corneal thickness, corneal volume, corneal eccentricity, and corneal asphericity (*r* = 0.87–0.98, all *P* < 0.001). Moderate interocular correlations were observed in whole corneal astigmatism (*r* = 0.78) and posterior corneal astigmatism (*r* = 0.73). ICC between the right and left eyes was 0.94–0.98 for anterior and posterior corneal curvatures, corneal diameter, corneal thickness and corneal volume, 0.80–0.88 for corneal eccentricity and asphericity, and 0.73–0.79 for corneal astigmatism (all *P* < 0.001). Bland–Altman plot showed that the 95% limit of agreement between both eyes was narrow and symmetric in most of the corneal biometrics, suggesting strong interocular agreements in these corneal biometrics. In conclusion, significant interocular symmetry of corneal biometrics is observed in Chinese myopia patients. Extra attention should be paid to patients with interocular corneal asymmetry.

## Introduction

Interocular symmetry of the cornea (ISC) in terms of agreement of corneal shape between the right eyes and left eyes has been observed in normal subjects^1^. In previous studies, interocular agreement of corneal biometrics such as corneal curvature, central corneal thickness and corneal elevation has been reported^1–3^. In corneal ectasia diseases, such as keratoconus, significant change of ISC has been reported^4,5^. Keratoconus is a bilateral but asymmetric non-inflammatory corneal disease with progressive corneal steeping and thinning, which can lead to increased myopia, irregular astigmatism and poor visual acuity^6^. Early detection and screening of keratoconus suspects before corneal refractive surgery is essential for the prevention of postoperative corneal ectasia. It has been shown that the corneal shape is significantly different between the right eyes and left eyes in keratoconus patients^4^. Therefore, change of ISC in myopic eyes before corneal refractive surgery may indicate an early sign of keratoconus^7,8^. Moreover, ISC is also important for the design of biosynthetic corneal substitutes^1^. With the regards of applying ISC in keratoconus diagnosis and corneal substitutes design, it is essential to know the normal range of the ISC. However, there are only few studies reporting the normal range of ISC in myopic eyes^9,10^. In the Shanghai High Myopia Study that included 202 cases of bilateral high myopia, researchers found good interocular symmetry in axial length, fixation, and magnitude of corneal astigmatism (ICC: 0.650–0.929), but interocular symmetry of optic disc tilt, rotation, and axis of corneal astigmatism was poor (ICC: 0.328–0.445)^9^. In another study, Myrowitz EH et al. evaluated the ISC in 121 patients before elective laser vision correction and found high interocular symmetry in simulated keratometry, minimum corneal thickness and posterior corneal elevation (*r*: 0.72–0.95)^10^. However, the sample size in these previous studies was relatively small, with only one statistical index (ICC or *r*) to evaluate the ISC. In the present study, we demonstrated the ISC of various corneal biometrics in a large number of myopic eyes before refractive surgery using multiple statistical indices.

## Results

A total of 13,288 myopia eyes of 6644 patients were recruited from five ophthalmic centers, including 2806 females (42.23%) and 3,838 males (57.77%). The average age was 25.12 ± 5.44 years. The mean SE was − 5.07 ± 1.95 D in the right eyes, and − 4.88 ± 1.95D in the left eyes. Demographics of the patients in the five ophthalmic centers were summarized in Table 1.

**Table 1:** Demographic data of patients by different ophthalmic centers.

Demographics	Ophthalmic centers						
	CD	GZ	HK	SY	WH	Pooled	P-Value*
Patients (n, %)	1153 (17.35%)	1878 (28.27%)	277 (4.17%)	1987 (29.91%)	1349 (20.30%)	6644 (100%)	N/A
**Age (years)**							
Mean ± SD	24.20 ± 5.50	26.81 ± 5.58	23.98 ± 4.91	23.92 ± 5.16	25.53 ± 5.04	25.12 ± 5.44	< 0.0001
Range	18–40	18–40	18–38	18–40	18–40	18–40	N/A
**Gender**							
Female (n, %)	415 (35.99%)	928 (49.41%)	98 (35.38%)	691 (34.78%)	674 (49.96%)	2806 (42.23%)	0.220
Male (n, %)	738 (64.01%)	950 (50.59%)	179 (64.62%)	1296 (65.22%)	675 (50.04%)	3838 (57.77%)	0.220
**SE (D)**							
OD, mean ± SD	− 5.00 ± 1.82	− 5.08 ± 2.18	− 5.72 ± 2.65	− 4.85 ± 1.70	− 5.30 ± 1.87	− 5.07 ± 1.95	< 0.0001
OD, range	− 18.38 to − 1.13	− 22.50 to − 0.63	− 22.00 to − 1.00	− 11.25 to − 0.75	− 20.38 to − 0.88	− 22.50 to − 0.63	N/A
OS, mean ± SD	− 4.80 ± 1.83	− 4.92 ± 2.12	− 5.44 ± 2.54	− 4.69 ± 1.75	− 5.11 ± 1.91	− 4.89 ± 1.95	< 0.0001
OS, range	− 17.13 to − 0.75	− 18.25 to − 0.75	− 17.38 to − 0.50	− 11.63 to − 0.50	− 19.00 to − 0.50	− 19.00 to − 0.50	N/A

*SD* standard deviation; *SE* spherical equivalent; *D* diopter; *GZ* Guangzhou Aier Eye Hospital; *SY* Shenyang Aier Eye Hospital; *CD* Chengdu Aier Eye Hospital; *WH* Wuhan Aier Eye Hospital; *HK* Hankou Aier Eye Hospital.

*Comparison among the five ophthalmic centers using Kruskal–Wallis test.

Corneal biometrics in both eyes, interocular difference and correlation coefficients of the corneal biometrics were shown in Table 2. It appeared that mean interocular difference in most of the corneal metrics was clinically negligible, except for the axis of KA and PCA. Significantly strong interocular correlations were observed in SimK, PCC, WTW, PA, PT, CV (3 mm, 5 mm, 7 mm), anterior and posterior corneal eccentricity and asphericity (*r* = 0.87–0.98, all *P* < 0.001). Moderate interocular correlations were observed in KA magnitude (*r* = 0.78) and PCA magnitude (*r* = 0.73).

**Table 2:** Summary of the corneal biometrics in both eyes.

Parameters	OD		OS		OD minus OS		*r* (95% CI)*	P*
	Mean ± SD	95% CI	Mean ± SD	95% CI	Mean ± SD	95% CI		
**Refraction**								
SE (D)	− 5.07 ± 1.95	− 5.12, − 5.02	− 4.89 ± 1.95	− 4.94, − 4.84	− 0.18 ± 0.90	− 0.20, − 0.16	0.89 (0.89, 0.90)	< 0.001
Cylinder (D)	− 0.75 ± 0.61	− 0.76, − 0.73	− 0.81 ± 0.64	− 0.83, − 0.79	0.07 ± 0.51	0.06, 0.08	0.65 (0.64, 0.67)	< 0.001
Axis(degree)	64.38 ± 68.77	62.63, 66.08	88.61 ± 74.29	86.76, 90.45	− 23.89 ± 107.68	− 26.02, − 20.91	0.04 (0.03, 0.08)	< 0.001
**Whole cornea**								
Sim K1 (D)	42.55 ± 1.34	42.52, 42.58	42.49 ± 1.34	42.46, 42.52	0.06 ± 0.32	0.06, − 0.07	0.97 (0.97, 0.97)	< 0.001
Sim K2 (D)	43.64 ± 1.43	43.60, 43.67	43.67 ± 1.44	43.63, 43.70	− 0.03 ± 0.38	− 0.04, − 0.02	0.96 (0.96, 0.96)	< 0.001
Sim Km (D)	43.09 ± 1.35	43.06, 43.13	43.08 ± 1.35	43.05, 43.11	0.02 ± 0.29	0.01, 0.02	0.98 (0.97, 0.98)	< 0.001
KA (D)	1.09 ± 0.61	1.07, 1.10	1.18 ± 0.63	1.16, 1.19	− 0.09 ± 0.40	− 0.10, − 0.08	0.78 (0.77, 0.79)	< 0.001
Flat axis (degree)	85.26 ± 78.15	83.38, 87.14	94.64 ± 78.93	92.74, 96.54	− 9.38 ± 127.54	− 12.45, − 6.32	− 0.16 (− 0.19, − 0.14)	< 0.001
WTW (mm)	11.65 ± 0.38	11.64, 11.66	11.66 ± 0.38	11.65, 11.67	− 0.01 ± 0.11	− 0.01, 0.00	0.96 (0.96, 0.96)	< 0.001
Pachy apex (μm)	543.83 ± 28.51	543.1, 544.5	544.56 ± 28.42	543.9, 545.2	− 0.73 ± 7.92	− 0.93, − 0.54	0.96 (0.96, 0.96)	< 0.001
Pachy thinnest (μm)	539.62 ± 28.62	538.9, 540.3	540.21 ± 28.50	539.5, 540.9	− 0.59 ± 8.17	− 0.79, − 0.39	0.96 (0.96, 0.96)	< 0.001
CV-3 mm (μm^3^)	3.93 ± 0.21	3.93, 3.94	3.93 ± 0.21	3.93, 3.94	− 0.00 ± 0.01	0.00, 0.00	0.94 (0.94, 0.94)	< 0.001
CV-5 mm (μm^3^)	11.54 ± 0.59	11.52, 11.55	11.54 ± 0.59	11.53.11.56	− 0.01 ± 0.17	− 0.01, 0.00	0.96 (0.95, 0.96)	< 0.001
CV-7 mm (μm^3^)	24.85 ± 1.26	24.81, 24.88	24.86 ± 1.26	24.83, 24.89	− 0.01 ± 0.37	− 0.02, 0.00	0.95 (0.95, 0.96)	< 0.001
**Anterior cornea**								
Eccentricity	0.53 ± 0.13	0.53, 0.54	0.54 ± 0.13	0.53, 0.54	0.00 ± 0.07	0.00, 0.00	0.87 (0.86, 0.87)	< 0.001
Asphericity	− 0.32 ± 0.12	− 0.32, − 0.32	− 0.32 ± 0.13	− 0.33, − 0.32	0.00 ± 0.06	0.00, 0.01	0.87 (0.86, 0.87)	< 0.001
**Posterior cornea**								
K1 (D)	− 6.10 ± 0.22	− 6.11, − 6.10	− 6.08 ± 0.22	− 6.09, − 6.08	− 0.02 ± 0.07	− 0.02, − 0.02	0.95 (0.94, 0.95)	< 0.001
K2 (D)	− 6.44 ± 0.25	− 6.45, − 6.43	− 6.44 ± 0.25	− 6.45, − 6.44	0.00 ± 0.09	0.00, 0.00	0.94 (0.94, 0.94)	< 0.001
Km (D)	− 6.27 ± 0.23	− 6.28, − 6.27	− 6.26 ± 0.23	− 6.27, − 6.26	− 0.01 ± 0.06	− 0.01, − 0.01	0.96 (0.96, 0.96)	< 0.001
PCA (D)	0.34 ± 0.14	0.33, 0.34	0.36 ± 0.14	0.36, 0.36	− 0.02 ± 0.10	− 0.03, − 0.02	0.73 (0.72, 0.74)	< 0.001
Flat axis (degree)	68.10 ± 78.80	66.21, 70.00	114.17 ± 78.72	112.3, 116.1	− 46.07 ± 123.57	− 49.04, − 43.09	− 0.08 (− 0.11, − 0.06)	< 0.001
Eccentricity	0.48 ± 0.16	0.48, 0.49	0.49 ± 0.17	0.48, 0.49	− 0.00 ± 0.08	0.00, 0.00	0.89 (0.88, 0.89)	< 0.001
Asphericity	− 0.32 ± 0.14	− 0.32, − 0.31	− 0.32 ± 0.15	− 0.32, − 0.32	0.00 ± 0.09	0.00, 0.01	0.89 (0.88, 0.89)	< 0.001

*SD* standard deviation; *CI* confidence interval; *SE* spherical equivalent; *D* diopter; *KA* keratometric astigmatism; *WTW* white-to-white; *CV* corneal volume; *PCA* posterior corneal astigmatism.

*Correlation coefficient and p value of the Spearman’s correlation test.

ICC and 95% LoA of the corneal biometrics between the right and left eyes were presented in Table 3. The ICC was 0.94–0.98 for SimK, PCC, WTW, PA, PT, and CV (3 mm, 5 mm, 7 mm), 0.80–0.88 for anterior and posterior corneal eccentricity and asphericity, and 0.73–0.79 for KA and PCA magnitudes (all *P* < 0.001). Bland–Altman plot showed that the 95% LoA between both eyes was narrow and symmetric in most of the corneal biometrics, suggesting strong agreements between the right and left eyes in these corneal biometrics, except for the axis of KA and PCA. The right-to-left ratios of the corneal biometrics were shown in Table 3, and the correlation coefficients between these ratios were presented in Table 4. For most of the corneal biometrics, the right-to-left ratios were within 0.99–0.12. As we can see, the right-to-left ratios of PA were strongly correlated with the right-to-left ratios of PT (*r* = 0.879). The right-to-left ratios of PA and PT were strongly correlated with the right-to-left ratios of CV 5 mm and CV 7 mm (*r* = 0.839–0.906), and were moderately correlated with the right-to-left ratios of CV 3 mm (*r* = 0.703–0.749).

**Table 3:** Summary of the ICC and LOA between the both eyes and the right-to-left ratio in different corneal biometrics.

Parameters	ICC			95% LOA			OD/OS ratio	
	ICC	95% CI	*p*	Lower limit	Upper limit	*P*	Mean ± SD	95% CI
**Refraction**								
SE (D)	0.89	(0.89, 0.90)	< 0.001	− 1.95	1.59	< 0.001	1.08 ± 0.39	(1.07,1.09)
Cylinder (D)	0.69	(0.68, 0.70)	< 0.001	− 0.92	1.06	< 0.001	0.96 ± 0.74	(0.94,0.98)
Axis (degree)	− 0.10	(− 0.12, − 0.07)	1.000	− 234.94	187.15	< 0.001	3.23 ± 9.11	(2.98,3.49)
**Whole cornea**								
Sim K1 (D)	0.97	(0.97, 0.97)	< 0.001	− 0.57	0.69	< 0.001	1.00 ± 0.01	(1.00,1.00)
Sim K2 (D)	0.97	(0.96, 0.97)	< 0.001	− 0.77	0.71	< 0.001	1.00 ± 0.01	(1.00,1.00)
Sim Km (D)	0.98	(0.98, 0.98)	< 0.001	− 0.55	0.58	< 0.001	1.00 ± 0.01	(1.00,1.00)
KA (D)	0.79	(0.79, 0.80)	< 0.001	− 0.88	0.69	< 0.001	1.04 ± 0.88	(1.02,1.06)
Flat axis (degree)	− 0.32	(− 0.34, − 0.30)	1.000	− 259.35	240.59	< 0.001	0.22 ± 1.05	(0.20,0.25)
WTW (mm)	0.96	(0.96, 0.96)	< 0.001	− 0.21	0.20	< 0.001	1.00 ± 0.01	(1.00,1.00)
Pachy apex (μm)	0.96	(0.96, 0.96)	< 0.001	− 16.25	14.79	< 0.001	1.00 ± 0.02	(1.00,1.00)
Pachy thinnest (μm)	0.96	(0.96, 0.96)	< 0.001	− 16.6	15.42	< 0.001	1.00 ± 0.02	(1.00,1.00)
CV-3 mm (μm^3^)	0.94	(0.94, 0.95)	< 0.001	− 0.14	0.14	0.004	1.00 ± 0.02	(1.00,1.00)
CV-5 mm (μm^3^)	0.96	(0.96, 0.96)	< 0.001	− 0.35	0.34	0.013	1.00 ± 0.02	(1.00,1.00)
CV-7 mm (μm^3^)	0.96	(0.95, 0.96)	< 0.001	− 0.74	0.72	0.011	1.00 ± 0.02	(1.00,1.00)
**Anterior cornea**								
Eccentricity	0.86	(0.85, 0.86)	< 0.001	− 0.14	0.14	0.004	1.02 ± 0.69	(1.00,1.04)
Asphericity	0.87	(0.86, 0.88)	< 0.001	− 0.12	0.13	< 0.001	1.01 ± 0.36	(1.00,1.00)
**Posterior cornea**								
K1 (D)	0.95	(0.95, 0.95)	< 0.001	− 0.16	0.12	< 0.001	1.00 ± 0.01	(1.00,1.00)
K2 (D)	0.94	(0.94, 0.95)	< 0.001	− 0.17	0.17	0.010	1.00 ± 0.01	(1.00,1.00)
Km (D)	0.97	(0.96, 0.97)	< 0.001	− 0.13	0.11	< 0.001	1.00 ± 0.01	(1.00,1.00)
PCA (D)	0.73	(0.72, 0.74)	< 0.001	− 0.22	0.18	< 0.001	0.99 ± 0.46	(1.00,1.00)
Flat axis (degree)	− 0.23	(− 0.25, − 0.21)	1.000	− 288.26	196.13	< 0.001	15.07 ± 77.48	(13.21,16.94)
Eccentricity	0.88	(0.88, 0.89)	< 0.001	− 0.16	0.15	0.065	1.02 ± 1.23	(1.00,1.05)
Asphericity	0.80	(0.79, 0.81)	< 0.001	− 0.18	0.18	0.003	1.02 ± 0.68	(1.01,1.04)

*ICC* intraclass correlation coefficient; *LOA* limit of agreement; *CI* confidence interval; *SD* standard deviation; *SE* spherical equivalent; *D* diopter; *KA* keratometric astigmatism; *WTW* white-to-white; *CV* corneal volume; *PCA* posterior corneal astigmatism.

**Table 4:** Spearman’s correlation coefficient (*r*) between the right-to-left ratios of different corneal biometrics.

Parameters	Sim Km	KA	WTW	Pachy Apex	Pachy Thin	CV-3 mm	CV-5 mm	CV-7 mm	Ecc A	Asph A	Km B	PCA	Ecc B	Asph B
Sim Km		0.151	− 0.022	0.077	0.087	0.059	0.072	0.092	0.279	0.275	**0.433**	0.082	0.077	0.068
KA	0.151		0.008	0.053	0.052	0.048	0.06	0.062	0.159	0.177	0.102	**0.412**	0.041	0.043
WTW	− 0.022	0.008		− 0.017	− 0.002	− 0.018	− 0.018	− 0.013	− 0.008	− 0.015	− 0.003	− 0.003	0.012	− 0.001
Pachy Apex	0.077	0.053	− 0.017		**0.879**	**0.749**	**0.906**	**0.882**	0.09	0.095	0.236	0.022	0.033	0.055
Pachy Thin	0.087	0.052	− 0.002	**0.879**		**0.703**	**0.857**	**0.839**	0.098	0.1	0.288	0.025	0.065	0.074
CV-3 mm	0.059	0.048	− 0.018	**0.749**	**0.703**		**0.743**	**0.733**	0.063	0.068	0.257	0.03	0.061	0.069
CV-5 mm	0.072	0.06	− 0.018	**0.906**	**0.857**	**0.743**		**0.919**	0.075	0.081	**0.382**	0.052	0.089	0.106
CV-7 mm	0.092	0.062	− 0.013	**0.882**	**0.839**	**0.733**	**0.919**		0.063	0.067	**0.446**	0.062	0.095	0.105
Ecc A	0.279	0.159	− 0.008	0.09	0.098	0.063	0.075	0.063		**0.928**	0.129	0.083	0.202	0.225
Asph A	0.275	0.177	− 0.015	0.095	0.1	0.068	0.081	0.067	**0.928**		0.128	0.094	0.213	0.25
Km B	**0.433**	0.102	− 0.003	0.236	0.288	0.257	**0.382**	**0.446**	0.129	0.128		0.193	**0.304**	0.287
PCA	0.082	**0.412**	− 0.003	0.022	0.025	0.03	0.052	0.062	0.083	0.094	0.193		0.048	0.092
Ecc B	0.077	0.041	0.012	0.033	0.065	0.061	0.089	0.095	0.202	0.213	**0.304**	0.048		**0.773**
Asph B	0.068	0.043	− 0.001	0.055	0.074	0.069	0.106	0.105	0.225	0.25	0.287	0.092	**0.773**	

Correlations with an *r* > 0.3 and a *p* < 0.05 are highlighted in bold.

*SE* spherical equivalent; *KA* keratometric astigmatism; *WTW* white-to-white; Thin, thinnest point; *CV* corneal volume; *ECC A* anterior corneal eccentricity; *Asph A* anterior corneal asphericity; *Km B* mean posterior corneal curvature; *PCA* posterior corneal astigmatism; *ECC B* posterior corneal eccentricity; *Asph B* posterior corneal asphericity.

## Discussion

The current study evaluated the interocular symmetry of corneal biometrics in myopic patients from five ophthalmic centers at different areas of Mainland China. There were strong interocular correlations and excellent interocular agreements in most the corneal biometrics, suggesting significant interocular symmetry in corneal morphology. Our results were consistent with a previous study in which the ISC in 3,835 subjects were investigated by Durr et al. using Orbscan II. In that study, there were significant interocular symmetry in corneal biometrics including corneal curvature, elevation, thickness, volume and WTW^1^. However, the upper and lower limits of ISC was not determined by Durr et al^1^. In the present study, the normal ranges of ISC are determined by the 95% LoA, which are potentially useful in the screening of keratoconus and design of corneal substitutes.

Accurate preoperative measurement of corneal biometrics is important for the success of refractive surgery. Routine preoperative screening of keratoconus also relies on the corneal biometrics. While the current screening systems of keratoconus consider the patients’ eyes as individual ones, the interactions between the both eyes are usually neglected. As a matter of fact, interocular symmetry of the corneal biometrics is also important for screening pathologies of the cornea. Rabinowitz et al. proposed that interocular asymmetry of central corneal power should be included as a criterion for keratoconus diagnosis^11^. Previous studies have shown that interocular symmetry of many corneal biometrics, such as corneal curvature, corneal thickness and corneal elevation, is significantly lower in patients with unilateral or bilateral keratoconus compared to normal subjects^12–14^. Keratometric asymmetry, topometric indices, and elevation differences (maximum-minimum) on both the anterior and posterior surfaces were statistically different in patients with unilateral keratoconus^15^. Corneal topographical parameters including surface regularity index, irregular astigmatism index and corneal eccentricity index were shown to be asymmetrical between the both eyes of patients with bilateral keratoconus^12^. Maria et al. also reported a greater interocular asymmetry in corneal thickness and posterior corneal elevation in keratoconus patients compared to normal subjects^13^.

China has the largest number of myopic patients in the world, and refractive surgery is becoming a popular treatment for myopia correction with dramatical increase of demands in China. Thus, it is of clinical importance to investigate the interocular symmetry in Chinese myopic adults, the largest population of refractive surgery candidates worldwide, to assist in preoperative screening of keratoconus. We first investigated the interocular difference of the corneal biometrics by subtracted the values of the left eyes from the right eyes. We found that the mean interocular difference in most of the corneal biometrics was close to zero. For example, the mean interocular difference was 0.06D for SimK1, − 0.03D for SimK2 and 0.02 for SimKm. In a previous study, Henriquez et al. reported that the mean interocular difference in corneal curvature on the flat axis and the steep axis was 0.29 ± 0.22D, 0.33 ± 0.31D respectively in normal subject and 2.73 ± 3.31D, 3.82 ± 4.18D respectively in patients with bilateral keratoconus^13^. The higher values of mean interocular difference of corneal curvature in normal subjects in Henriquez’s study was due to the calculating method in which the interocular difference was determined by subtracting the lowest value from the highest value for each variable. For the interocular difference of SimKm, we also demonstrated a 95% LoA of − 0.55D to 0.58D, suggesting extra attention should be paid to patients with an interocular difference of SimKm beyond this range. Whether myopic patients with larger interocular difference of the corneal biometrics are prone to developing postoperative ectasia are unclear. However, a longer and more frequent follow-up after corneal refractive surgery may be necessary for those “outliers”.

The interocular correlations and agreements of the corneal biometrics were also analyzed in the present study. The interocular correlation coefficients and ICCs were ≥ 0.8 for most of the corneal biometrics. For the SimK, PCC, WTW, CTA, CTT, and CV (3 mm, 5 mm, 7 mm), the interocular correlation coefficients and ICCs were ≥ 0.94, which meant strong agreements between right and left eyes. The results were consistent with a previous study in which the interocular correlation coefficient was 0.90 for SimKm and 0.95 for PA^10^.

Despite significant interocular symmetry in most of the corneal biometrics, the KA and PCA (especially their axis) are less symmetrical between the right and left eyes in the current study. The correlation coefficient was 0.78 for KA magnitude and 0.73 for PCA magnitude, and the ICC was 0.79 for KA magnitude and 0.73 for PCA magnitude. The 95% LoA was − 0.88D to 0.69D for KA magnitude. The findings were consistent with a previous study^9^. Interocular symmetry of the KA and PCA axis was very poor in our study, and the results were similar when we flipped the KA and PCA axis of the left eye horizontally across the vertical meridian. These findings were inconsistent with a previous study showing excellent interocular agreement in axis of corneal astigmatism using mirrored image of the left eye topography^1^. It is difficult to explain why the corneal astigmatism is less symmetrical between the both eyes in our study. Previous studies have shown that 1–15% of 6–13 years old children have an interocular difference of more than 1.00 D in corneal astigmatism^16–18^. The reason of asymmetry corneal astigmatism in children may be due to an asymmetric working distance between the two eyes, which may affect the peripheral retina imaging^19^. Taken together, these findings make the interocular symmetry of KA and PCA less valuable in screening keratoconus.

In the current study, there were correlations between the right-to-left ratios of corneal thickness biometrics and those of the corneal volume biometrics, with the strongest correlation between the right-to-left ratios of PA and CV 5 mm (*r* = 0.906). These findings indicate that the interocular differences in corneal thickness are associated with interocular differences in corneal volume. In a previous study, eyes with keratoconus were shown to have significantly thinner corneal thickness and smaller corneal volume compared to normal eyes^20,21^. Surprisingly, the correlations between the right-to-left ratios of corneal thickness was stronger than those of the corneal volume. We are not sure of the mechanisms underlying this finding, although we speculate that the interocular differences of CV in the central 3 mm, 5 mm and 7 mm corneal area are not highly parallel to each other. We also observed weak correlations between the right-to-left ratios of SimK and PCC, and between the right-to-left ratios of KA and PCA, suggesting that the interocular differences in the anterior cornea were not parallel to those of the posterior cornea.

In conclusion, interocular symmetry of various corneal biometrics in myopia is investigated using a large number of multicenter data in the current study. Our findings provide meaningful evidences of interocular agreement in myopic patients, enabling us to better understand the relationships between the right and left eyes in cornea morphology. A better understanding of the interocular symmetry will also help with the keratoconus screening systems, and allows better design of corneal substitutes.

## Methods

### Participants

A total of 13,288 eyes of 6644 myopic patients were recruited in this multicenter study. The patients were from five ophthalmic centers, including Guangzhou Aier Eye Hospital (GZ; 113.2°E 23.1°N, altitude 43.4 m), Shenyang Aier Eye Hospital (SY; 123.4°E 41.8°N, altitude 51.0 m), Chengdu Aier Eye Hospital (CD; 104.0°E 30.7°N, altitude 505.9 m), Wuhan Aier Eye Hospital (WH; 114.2°E 30.4°N, altitude 23.3 m), and Hankou Aier Eye Hospital (HK; 114.1°E 30.4°N, altitude 27.6 m). The study was approved by the Institutional Review Board of every hospital (GZ, SY, CD, WH and HK) and is in agreement with the Declaration of Helsinki. Since only review of medical records was conducted and no individual patient could be identified from the data, informed consent was waived by the IRBs^22^. Digital medical records of patients who underwent ocular assessment before refractive surgery for myopia between 2017 and 2019 were reviewed, and patients meeting the inclusion criteria were included. Both of the right eye and the left eye of the patients were included for analysis. Inclusion criteria were myopic patients with a spherical equivalent (SE) ≤ − 0.50 D and good quality Scheimpflug scans in both eyes, a stable refractive error (≤ 0.50 D of refractive error change in the past 2 years). Exclusion criteria were coexisting corneal diseases, keratoconus, forme fruste keratoconus, severe dry eye, non-axial myopia (such as those caused by spherophakia), previous ocular trauma or surgery, uveitis, glaucoma, wearing contact lenses within the previous 2 weeks, age younger than 18 years (unstable refraction) or older than 40 years (to reduce the effects of the crystal lens on refraction)^22^.

### Examinations

All of the eyes underwent thorough ophthalmic examinations including best-corrected visual acuity (BCVA), intraocular pressure (IOP), cycloplegic and manifest refraction, anterior segment examination by slit-lamp, corneal topography and Pentacam measurements. Clinical data of the eyes were retrieved from an electronic medical record database. The spherical equivalent (SE) was defined as “spherical error + 1/2 cylindrical error”.

The corneal biometrics were measured with Pentacam by experienced technicians as previously described^22^. The Pentacam instrument (Oculus GmbH, Wetzlar, Germany) was calibrated regularly on a weekly basis. Proper positioning of the patients and even distribution of the tear film were assured before Pentacam measurement. The instrument automatically captured 50 rotational Scheimpflug images of the cornea within 2 s. The anterior and posterior corneal radius within the central 3 mm area were measured. Simulated corneal curvature (SimK, K1 for the flat axis, K2 for the steep axis and Km for the mean curvature), keratometric astigmatism (KA), posterior corneal curvature (PCC, K1 for the flat axis, K2 for the steep axis and Km for the mean curvature) and posterior corneal astigmatism (PCA) were calculated as previously described^23,24^. The horizontal corneal diameter (white-to-white, WTW), corneal thickness at the apex (PA) and the thinnest point (PT), corneal volume (CV) within the 3 mm, 5 mm and 7 mm areas, anterior and posterior corneal eccentricity and asphericity were also obtained. The measurement was performed again if the patient’s eye blinked or the scan quality was poor. Only images covering at least 8.0 mm of the central corneal with the image quality labelled with ‘OK’ were accepted. Pentacam data of the eyes were retrieved from the machine and only results with image quality labelled with ‘OK’ were included^22^.

### Statistical analysis

Statistical analysis was performed using STATA software (version 15.0, stata, Inc.) A Kolmogorov–Smirnov (KS) test was used to evaluate normality of all variables. Data of age, SE, and the corneal biometrics were presented as mean ± standard deviation (SD). Interocular correlation was expressed as Spearman’s correlation coefficients (*r*), and interocular agreement was evaluated by intraclass correlation coefficients (ICC)^25^. Strength of the correlation/agreement was classified as “strong” if the *r* or ICC was ≥ 0.8, as “moderate” if the r or ICC was 0.60 to 0.79, as “weak” if the r or ICC was 0.40 to 0.59, and as “poor” if the *r* or ICC was less than 0.4. Interocular differences of the corneal biometrics were plotted against the averages of the both eyes using Bland–Altman plot^26^, and the 95% limit of agreement (LoA) was shown. We also calculated the right-to-left ratios of the corneal biometrics and the correlations between these ratios were analyzed using Spearman’s correlation test. *P* < 0.05 was considered to be statistically significant.

## Data Availability

The data used during the current study are available from the corresponding author on reasonable request.
